# The effect of extracellular vesicles derived from oral squamous cell carcinoma on the metabolic profile of oral fibroblasts

**DOI:** 10.3389/fmolb.2025.1492282

**Published:** 2025-02-21

**Authors:** Aleksandra Lipka, Tine M. Søland, Anni I. Nieminen, Dipak Sapkota, Trude M. Haug, Hilde K. Galtung

**Affiliations:** ^1^ Faculty of Dentistry, Institute of Oral Biology, University of Oslo, Oslo, Norway; ^2^ Institute for Molecular Medicine Finland, University of Helsinki, Helsinki, Finland

**Keywords:** oral cancer, oral squamous cell carcinoma, fibroblasts, phenotype, metabolic profile, extracellular vesicles, EV, OSCC

## Abstract

**Introduction:**

Oral cancer is one of the most common forms of head and neck cancers. Oral squamous cell carcinoma (OSCC) accounts for more than 90% of the oral malignancies. The molecular pathogenesis of OSCC is complex as it involves altered expression of specific genes and proteins, but also comprises changes in metabolic processes. It is suggested that extracellular vesicles (EVs) released by cancer cells may contribute to cancer development and metastasis by recruiting and changing phenotype of normal cells that surround the tumor.

**Methods:**

The aim of the project was to characterize the effect of OSCC EVs on the metabolic profile of normal oral fibroblasts (NOFs). Targeted liquid chromatography mass spectrometry metabolic profiling was performed on control cells and NOFs exposed to OSCC EVs for 24 and 48 h.

**Results:**

Analysis of detected metabolites revealed that OSCC EVs affected NOFs the most after 24 h of exposure. Among metabolites that were significantly altered at 24 h, pyruvate, ATP, UTP, coenzyme A, and dihydroxyacetone phosphate were upregulated, while fatty acids such as nervonic acid, linoleate, oleate, palmitoleic acid, and docosahexaenoic acid were downregulated. These findings were supported by Western blotting of pyruvate kinase M2 (PKM2) and aldolase, fructose-bisphosphate A (ALDOA).

**Conclusion:**

The metabolic pathways of glycolysis, citric acid cycle, and amino acid metabolism were enriched, suggesting that OSCC EVs cause phenotype switch in NOFs that may contribute to acquiring a pro-tumorigenic phenotype.

## 1 Introduction

Oral cancer, one of the most common form of head and neck cancers, has an annual incidence rate of ~380,000 and it holds the sixteenth position in malignancies worldwide (Chamoli et al., 2021). Oral squamous cell carcinoma (OSCC) accounts for more than 90% of the oral malignancies (Badwelan et al., 2023). OSCC development is a multistep process that seems to be a result of patients’ genetic predisposition, environmental factors, age and epigenetic modifications (Aghiorghiesei et al., 2022). The major risk factors for OSCC development are tobacco use, betel quid usage, and alcohol misuse (Warnakulasuriya, 2009). These factors may act individually or interdependently with multiplicative effect on the cancer risk (Lissowska et al., 2003; Subapriya et al., 2007). Usually, OSCC is preceded by oral potentially malignant disorders (OPMD), like oral leukoplakia, having the same etiological risk factors as OSCC (Bewley and Farwell, 2017).

It is becoming clear that the development and progression of cancer, including OSCC, involve not only genetic/epigenetic abnormalities in the cancer cells, but also require a mutual interplay between the tumor cells and adjacent stromal cells such as the cancer associated fibroblasts (CAFs), immune cells, and endothelial cells. This complex network of various cell types in the vicinity of the tumor is referred to as the tumor microenvironment (TME) (Wang et al., 2021). The cells in the TME may undergo reprogramming to provide a tumor-supportive milieu and produce signaling molecules that can for example, promote cancer cell proliferation and metastasis (Shan et al., 2021). CAFs are involved in several biological processes such as cancer cell proliferation and migration, angiogenesis, and extracellular matrix (ECM) remodeling, thereby contributing to tumor progression and metastasis. In some types of cancer, CAFs are considered a central component of the TME (Arima et al., 2023) and in OSCC their presence has been linked to increased tumor invasion and worse prognosis (Fujii et al., 2012; Liu et al., 2016). Due to the complex and varied TME composition, the crosstalk between its elements is also complex and multi-leveled. Within the TME, cells can communicate directly via cell-to-cell contact and adhesion molecules, electrical coupling through gap junctions, or indirectly through releasing cytokines, growth factors, and extracellular vesicles (EVs) (Dominiak et al., 2020).

EVs are a heterogeneous population of lipid bilayered vesicles, ranging in diameter from 30 to 10,000 nm. As naturally occurring and released by all cell types, EVs have emerged as an important player in intercellular communication, mainly through their ability to transfer their biological cargo of proteins, lipids, and nucleic acids to recipient cells (Valadi et al., 2007; Subra et al., 2010). The physiological function of EVs include regulation of various processes such as angiogenesis, cell survival, proliferation, and apoptosis (Li et al., 2022). However, EVs may also be involved in pathophysiological processes, and could be part of the background for disease onset and progression (Van Niel et al., 2018).

The molecular pathogenesis of OSCC is complex as it involves altered expression of specific genes and proteins, but also comprises changes in metabolic processes (Wei et al., 2011; Xie et al., 2012). In particular, the metabolome, reflecting the interactions between genome, microbiome, and environmental factors, is dynamic and diverse (Beger et al., 2016). In general, one of the most characteristic metabolic features of the cancer cells is increased glucose uptake and its altered metabolism. After metabolizing glucose to pyruvate (glycolysis), cancer cells switch from oxidative phosphorylation (OXPHOS) of pyruvate in the citric acid cycle (TCA) to fermentation of pyruvate into lactic acid. The latter is an anaerobic mechanism preferred by cancer cells even when adequate amounts of oxygen is present. Since it is less effective in energy production than OXPHOS, it must have other benefits (Gray et al., 2014). Recent studies have revealed that normal oral fibroblasts (NOFs) co-cultured with OSCC cells, change their metabolic phenotype into a pro-tumorigenic one. This change was characterized by increased rate of aerobic glycolysis, increased levels of reactive oxygen species and lactate, and overexpression of the lactate transporter MCT-4 in the NOFs (Zhang et al., 2020), termed the reverse Warburg effect. MCT-4 exports lactate out of the fibroblasts, making it available as fuel for cancer cells and at the same time acidifying the tumor environment. However, the mechanism of this fibroblast phenotype switch is not fully understood, nor is the nature of interaction between cancer cells, cells that constitute the TME, and normal cells in the tumor vicinity. We hypothesize that EVs derived from OSCC cells can affect normal oral cells and modify them towards a pro-tumorigenic phenotype. Therefore, the aim of the project was to characterize the effect of OSCC EVs on the metabolic profile of normal oral fibroblasts (NOFs).

## 2 Materials and methods

### 2.1 Cell cultures

Primary NOFs (a kind gift from Prof. D. Costea, University of Bergen, Norway; REK approval 2010/481), collected and cultured as described previously (Zhang et al., 2020; Sharma et al., 2023), were used in the current study. Isolation and use of these cells were approved by the Regional Committees for Medical Research Ethics Western Norway (REK-2010/481). The EVs were isolated from a commercially available human oral squamous cell carcinoma line (PE/CA-PJ49/E10; ECACC, Salisbury, United Kingdom) with a combination of cell media modification, ultrafiltration, and size exclusion chromatography (Guerreiro et al., 2018; 2020). The EVs were characterized using nano tracking analysis, transmission electron microscopy, Western blotting, and flow cytometry, and were found CD9 positive with a size between 60 and 140 nm.

NOFs were maintained in DMEM, high glucose, GlutaMAX™ Supplement medium (Thermo Fisher Scientific, Waltham, MA, United States) supplemented with 10% v/v fetal bovine serum (FBS) and 1% Penicillin/Streptomycin/Amphotericin solution (PSA; Corning Life Sciences, New York, NY, United States). NOFs were passaged by trypsinization at approximately 75%–80% confluence and grown under standard cell culture conditions in a humidified incubator at 37°C and 5% CO_2_/95% air. Following trypsinization, 300,000 cells/well were seeded into six-well plates. To reduce the exosome background, the FBS was replaced by exosome-depleted FBS (Thermo Fisher Scientific, Waltham, MA, United States). The day after seeding, 10^7^ OSCC-derived EVs (Guerreiro et al., 2018; 2020) were added to each well and incubated for 15 min, 24 h, and 48 h. NOFs in culture without added EVs were used as controls. For each treatment and time point, four technical replicates were used. At each time point supernatant was removed, and NOFs were washed twice with PBS. Then, ice-cold PBS (200 µL) and a scraper was used to harvest the cells that were subsequently pelleted (10 min; 13,000 rpm) and submitted for liquid chromatography-mass spectrometry (LC-MS) metabolic profiling (Metabolomics Unit, Institute for Molecular Medicine Finland FIMM).

### 2.2 Targeted LC-MS metabolic profiling and data analysis

Metabolites were extracted from pelleted cells using 400 µL of cold extraction solvent (Acetonitrile:Methanol:MQ; 40:40:20; Thermo Fisher Scientific, Waltham, MA, United States) and subsequently, samples were vortexed for 2 min and sonicated for 1 min (settings: sweep mode, frequency 37, power 60, no heating), followed by centrifugation at 14,000 rpm (4°C; 5 min). Supernatants were transferred into HPLC glass auto sampler vials. Next, 2 µL of sample were injected to Thermo Vanquish UHPLC coupled with Q-Exactive Orbitrap quadrupole mass spectrometer equipped with a heated electrospray ionization (H-ESI) source probe (Thermo Fisher Scientific, Waltham, MA, United States). A SeQuant ZIC-pHILIC (2.1 × 100 mm, 5-μm particle) column (Merck KGaA, Darmstadt, Germany) was used for chromatographic separation. The gradient elution was carried out with a flow rate of 0.100 mL/min using 20 mM ammonium hydrogen carbonate (pH 9.4) with 25% ammonium solution as mobile phase A and acetonitrile as mobile phase B. The gradient elution was initiated from 20% of mobile phase A and 80% of mobile phase B and maintained for 2 min, followed by mobile phase A gradually increasing up to 80% till 17 min, then mobile phase A decrease from 80% to 20% in 17.1 min, and 20% maintained up to 24 min. The temperature of the column oven and auto-sampler was set to 40°C ± 3°C and 5°C ± 3°C, respectively. MS was equipped with a heated electrospray ionization (HESI) source using polarity switching and following setting: resolution of 35,000, the spray voltages: 4250 V for positive and 3250 V for negative mode, the sheath gas: 25 arbitrary units (AU), and the auxiliary gas: 15 AU, sweep gas flow 0, capillary temperature: 275°C, S-lens RF level: 50.0. Instrument control was operated with the Xcalibur 4.1.31.9 software (Thermo Fisher Scientific, Waltham, MA, United States). The metabolite annotation was based on accurate mass m/z (5 ppm) and confirmed retention times from inhouse library kit MSMLS-1EA (Merck, Darmstadt, Germany). The peak integration was performed with the TraceFinder 4.1 software (Thermo Fisher Scientific, Waltham, MA, United States) using ICIS algorithm, threshold for 50,000, smoothing 7. The data quality was monitored throughout the run using QC sample (human serum) that was interspersed as every 10th sample throughout the run along with blank samples. The metabolite data were checked and filtered for peak quality (poor chromatograph), high variation, % relative standard deviation (%CV), in serum QC samples (acceptable<20%), blank carryover (acceptable <20%).

The obtained dataset was analysed with Metaboanalyst 5.0 (https://www.metaboanalyst.ca). Missing variables were replaced by LoDs (limit of detection). The values were normalised to total peak intensity, log-transformed and auto-scaled. Subsequently, statistical analyses (including fold-change analysis, t-test) were performed and multivariate clustering analysis was computed and visualized as a heatmap (distance measure using Euclidean, and clustering algorithm using ward.D). To provide an overall functional insight, over-representation analysis (ORA) as well quantitative enrichment analysis (QEA) was performed (Metaboanalyst 5.0; https://www.metaboanalyst.ca) for commonly detected metabolites within the SMPDB (The Small Molecule Pathway Database; https://www.smpdb.ca) and KEGG (Kyoto Encyclopedia of Genes and Genomes; https://www.kegg.jp) databases.

### 2.3 Western immunoblotting

Among detected metabolites, pyruvate and dihydroxyacetone phosphate differentiated the most and were associated with pathways that may be important in malignant transformation, and therefore were selected as validation targets. Western immunoblotting was performed to assess the levels of pyruvate kinase isozyme M2 (PKM2) and aldolase, fructose-bisphosphate A (ALDOA) expression, enzymes directly involved in pyruvate and dihydroxyacetone phosphate metabolism, respectively. To isolate proteins, the NOF cell pellet was lysed in 150 µL of CelLytic™ buffer (Merck KGaA, Darmstadt, Germany). Next, 10 µg of protein from each sample was electrophoresed through a 4%–12% Bolt™ Bis-Tris Plus gel (Thermo Fisher Scientific, Waltham, MA, United States) and transferred to a 0.45 µm Immobilon®-FL PVDF membrane (Merck KGaA, Darmstadt, Germany). Membranes with transferred proteins were blocked in casein blocking buffer (Merck KGaA, Darmstadt, Germany). To detect PKM2 and ALDOA, each blot was incubated in 1:5,000 dilution (in casein blocking buffer) of anti-PKM2 rabbit polyclonal (HPA029501, Merck KGaA, Darmstadt, Germany) or 1:500 dilution of anti-ALDOA rabbit polyclonals (HPA004177, Merck KGaA, Darmstadt, Germany) for 24 h at 4°C. As a loading control, mouse anti-GAPDH monoclonals (ab 9484, Abcam, Cambridge, United Kingdom) were used in 1:1,000 dilution, and as a positive control cell lysates from the Jurkat cell line (ACC 282, Leibniz Institute DSMZ, Leibniz, Germany) were used. Then, membranes were washed (3 × 5 min/1× TBST) and transferred into a 1:10,000 dilution (in casein blocking buffer) of each: goat anti-Rabbit IgG Alexa Fluor Plus 680 (Thermo Fisher Scientific, Waltham, MA, United States) and goat anti-Mouse IgG Alexa Fluor Plus 800 (Thermo Fisher Scientific, Waltham, MA, United States). After incubation with secondary antibodies for 1 h in room temperature (RT), PVDF membranes were washed (3 × 5 min/1× TBST) and then imaged with iBright system (Thermo Fisher Scientific, Waltham, MA, United States). Densitometry calculations were performed using ImageJ (https://imagej.net/ij/) and were normalized to GAPDH. Statistical significance was calculated using Student’s t-test.

## 3 Results

### 3.1 Exposure of OSCC EVs resulted in altered metabolic profile of NOF

**FIGURE 1:**
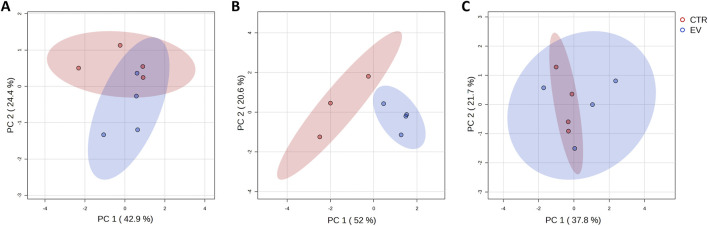
A Graphical representation of the first (PC 1) and second (PC 2) principal component displaying distribution of control (CTR, red) and EV-treated (EV, blue) samples collected at different time points: 15 min **(A)**, 24 h **(B)** and 48 h **(C)**.

Fibroblasts exposed to OSCC EVs and controls (fibroblasts without OSCC EV exposure) were submitted for metabolite extraction and targeted profiling of 461 metabolites. In summary, for the whole dataset, 141 common metabolite peaks were detected and the obtained data were used to characterize the metabolic profile of the analyzed cells. Initial data processing indicated that one of the control samples (control_2_24 h time point) was an outlier according to overall intensity and high amount of missing values and this sample was excluded from further analysis. Principle component analysis (PCA) for the remaining dataset (11 controls and 12 EV-treated samples from all time points) did not reveal explicit grouping of the samples based on the metabolic profiles. However, PCA performed for each time point separately (Figure 1) demonstrated that control and EV-exposed samples could be differentiated, especially at the 24 h time point (Figure 1B).

**FIGURE 2:**
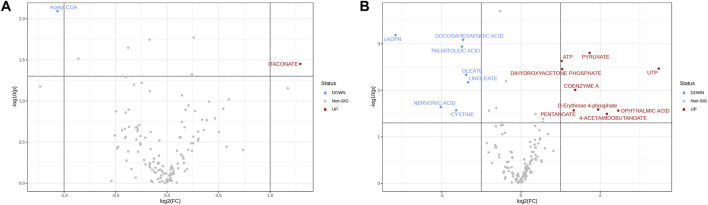
Volcano plots showing log2FC plotted against p-values for 15 min **(A)** and 24 h **(B)** time points. Dotted horizontal line indicates negative logarithmic p-value (0.05) cut-off. Dotted vertical lines indicate applied fold change (absolute value of log2FC ≥ 1) cut-off. Each point (dot) represents single metabolite expression value; blue (downregulated in EV-treated samples) and red (upregulated in EV-treated samples) represent the significantly dysregulated metabolites (p ≤ 0.05).

Analysis of the peak intensities of controls and EV-treated samples was performed to indicate potentially dysregulated metabolites. At 15 min, acetyl-CoA was upregulated and itaconate was downregulated (FC ≥ 2; p-value ≤ 0.05) in EV-treated samples (Figure 2A). At the 24 h time point 7 metabolites were downregulated and nine were upregulated in EV-treated samples (Figure 2B), while at 48 h no significantly dysregulated metabolites were detected (FC ≥ 2; p-value ≤ 0.05; data not shown).

**FIGURE 3:**
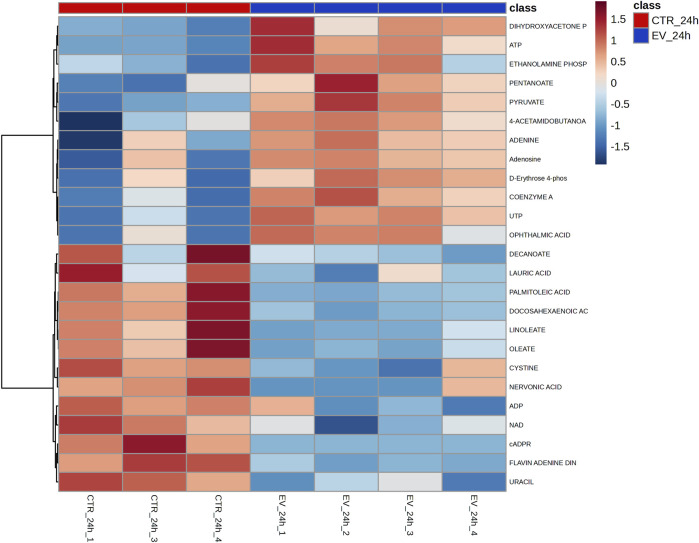
Heatmap of peak intensities data for the top-25 most dysregulated metabolites detected at the 24 h time point in control (CTR) and EV-exposed (EV) samples. Columns represent individual samples; rows represent each metabolite.

Among the dysregulated metabolites at 24 h, metabolites such as pyruvate (p = 0.0016), ATP (p = 0.0023), UTP (p = 0.0034), coenzyme A (p = 0.0098), and dihydroxyacetone phosphate (p = 0.0035) were significantly upregulated, while fatty acids such as nervonic acid (p = 0.02), linoleate (p = 0.01), oleate (p = 0.0045), palmitoleic acid (p = 0.001), and docosahexaenoic acid (p = 0.0008) were significantly downregulated in fibroblasts treated with EVs (Figure 2B). Overview of peak intensities for detected metabolites is presented in Figure 3.

### 3.2 Functional analysis

**FIGURE 4:**
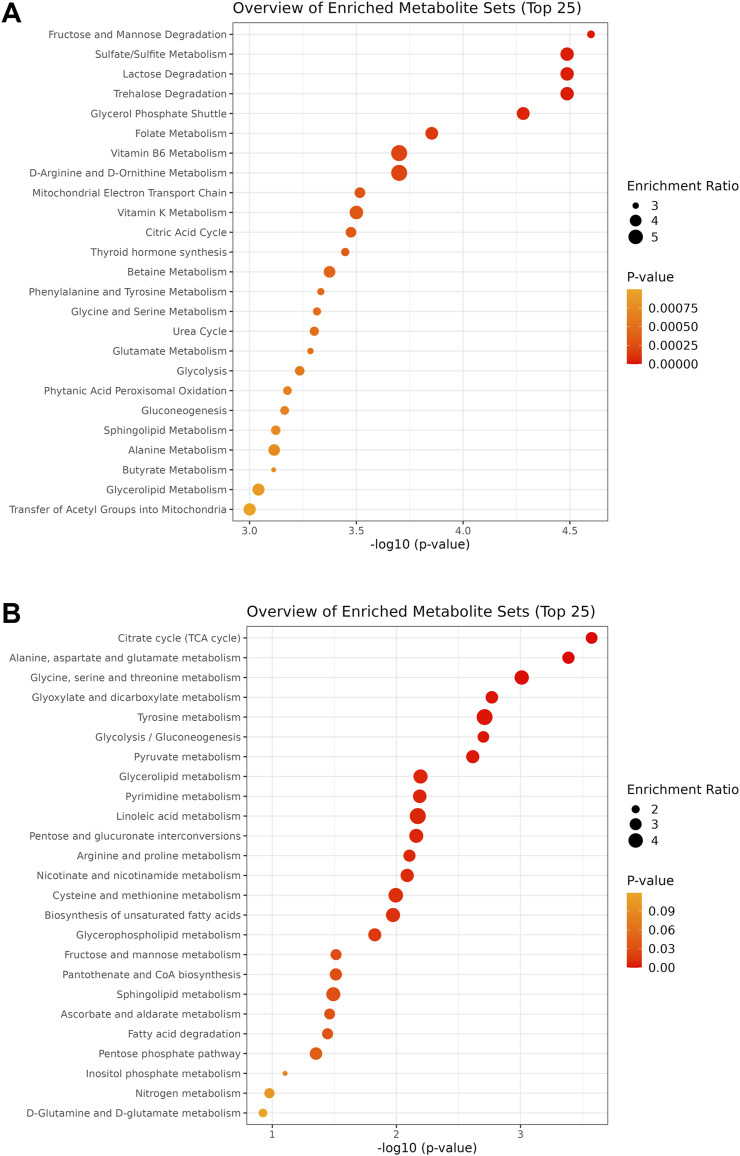
Charts of the pathways detected within the SMPDB **(A)** and KEGG **(B)** databases during quantitative enrichment analysis of the 24 h time point dataset. Circle size is proportional to the enrichment ratio. Logarithmic scale of p-value was calculated from peak intensities and the number of up and downregulated metabolites.

Functional analysis was performed for the samples collected at the 24 h time point. Quantitative enrichment analysis (QEA) indicated 83 pathways as significantly enriched within the SMPDB database. Among these, fructose and mannose degradation, sulfate/sulfite metabolism, and lactose degradation pathways were the most enriched (all at p = 0.00003; Figure 4A). Furthermore, QEA run within the KEGG database showed 23 significantly enriched pathways with the highest enrichment for citrate cycle (p = 0.0003), alanine, aspartate and glutamate metabolism (p = 0.0004), and glycine, serine, and threonine metabolism (p = 0.001) (Figure 4B). High enrichment score was also obtained for glycolysis (p = 0.0019) and pyruvate metabolism (p = 0.0024).

**FIGURE 5:**
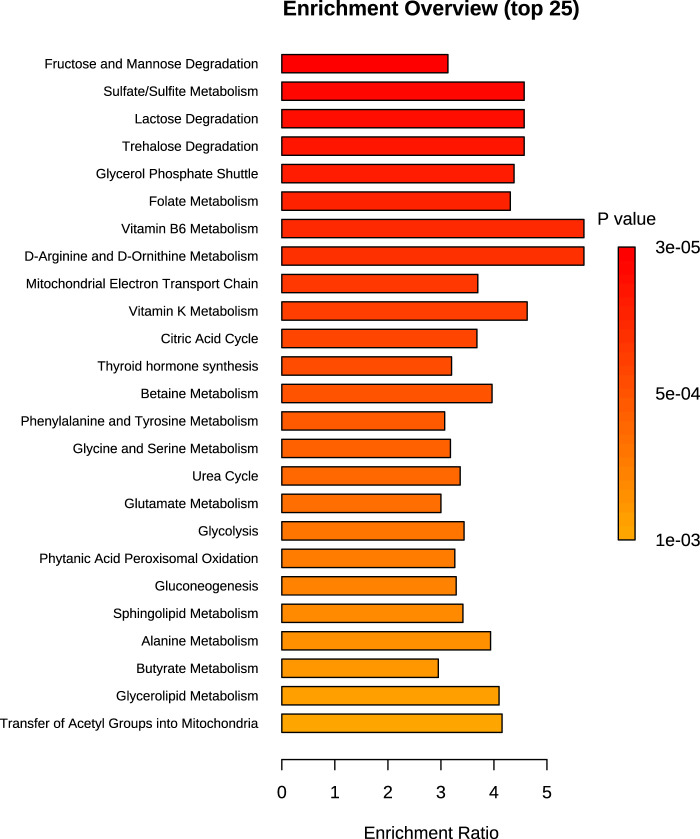
Chart of the top 25 SMPDB pathways detected during over-representation analysis of the 24 h time point dataset. Bar size is proportional to the enrichment ratio. Logarithmic scale of p-value was calculated from the number of metabolites that were enriched.

**FIGURE 6:**
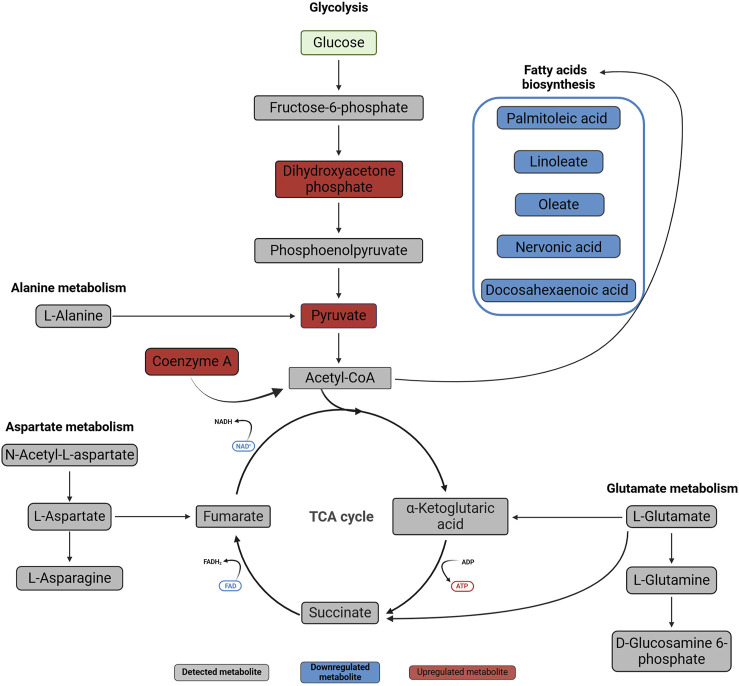
Metabolic changes in normal oral fibroblasts after exposure to OSCC EVs for 24 h. Metabolites present in the studied dataset are presented with names, downregulated metabolites are marked with blue and upregulated metabolites with red, while metabolites not significantly affected are in grey. The metabolites were linked to pathways using the Kyoto Encyclopedia of Genes and Genomes (KEGG) database (https://www.kegg.jp/kegg/pathway.html). Created with BioRender.com.

**FIGURE 7:**
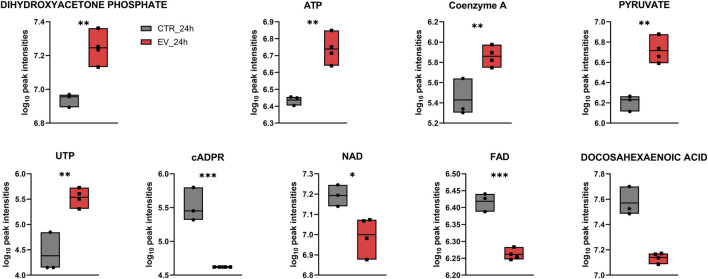
Box plots for individual metabolites detected at the 24 h time point that contributed to the enrichment of processes such as glycolysis, citric acid cycle, mitochondrial electron transport chain, and Warburg effect. * – p ≤ 0.05; ** – p ≤ 0.01; *** – p ≤ 0.001.

Over-representation analysis (ORA) indicated glutamate metabolism, Warburg effect and urea cycle among the most enriched pathways (Figure 5). In general, the QEA and ORA results indicate that EV treatment mostly affected pathways associated with sugar and amino acid metabolism. An overview of QEA and ORA results showed that some metabolites were associated with multiple pathways, such as glycolysis, citric acid cycle, mitochondrial electron transport chain, and Warburg effect (Figure 6). EV-treated fibroblasts were characterized by significantly upregulated dihydroxyacetone phosphate, ATP, coenzyme A, pyruvate, and UTP (Figure 7) all important intermediates in these pathways. Among downregulated metabolites, NAD, FAD, cADPR, and docosahexaenoic acid (Figure 7) were enriched in the pathways listed above.

### 3.3 Western blot analysis

**FIGURE 8:**
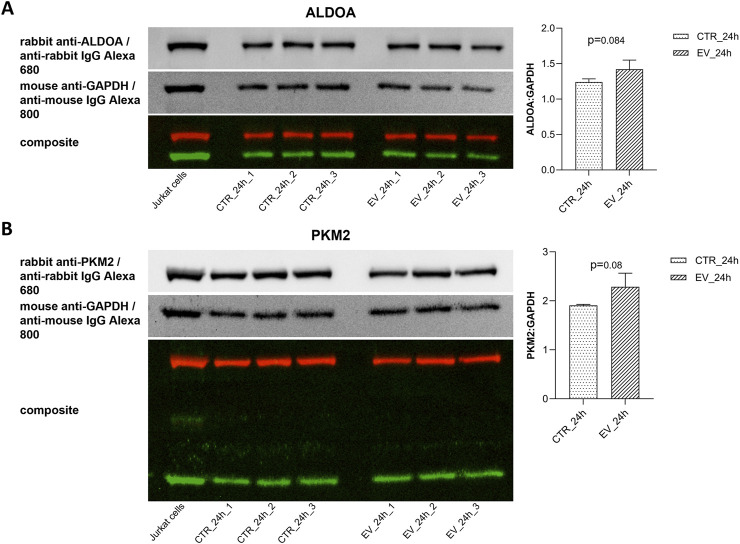
Western blots and bar diagrams of densitometric analysis of ALDOA **(A)** and PKM2 **(B)** expression in relative to GAPDH. Data are shown as mean values ± SD.

To validate results obtained with metabolic analysis, Western blot analyses (Figure 8) were performed for the enzymes directly involved in dihydroxyacetone phosphate and pyruvate metabolism, i.e., ALDOA and PKM2, respectively. Overexpression (14.5%) of ALDOA was found in NOFs exposed to OSCC EVs as compared to the controls (Figure 8A). Similarly, treatment with OSCC EVs resulted in an 19.8% increase in PKM2 expression (Figure 8B). However, the results were not statistically significant (0.05 < p < 0.1). Nevertheless, the direction of change and level of increase was comparable with the metabolic data.

## 4 Discussion

The present study explored the metabolic profile in normal oral fibroblasts (NOFs) exposed to EVs of OSCC origin. We identified dysregulation of several key metabolites (Figure 7) associated with pathways (Figure 6) that may be important for metabolic reprogramming of NOFs into a pro-tumorigenic phenotype.

Among the metabolites that were significantly dysregulated, an increase in pyruvate level was seen in NOFs treated with EVs for 24 h, and as pyruvate is the end-product of glycolysis, it may indicate dysregulation of this major metabolic pathway. Pyruvate can be oxidized and further processed in the TCA cycle, or alternatively it can undergo fermentation to lactic acid (Gray et al., 2014). Our results suggests that exposure of NOFs to OSCC EVs may have caused a switch in NOFs from pyruvate oxidation to fermentation. The pathway analyses indicate that besides glycolysis, the most enriched pathways in NOFs after exposure to OSCC EVs include pyruvate metabolism and Warburg effect, which is manifested by pyruvate fermentation and lactate accumulation during glucose metabolism even in the presence of abundant oxygen (Warburg, 1956). In the cancer cells the majority of glucose is metabolized through fermentation (66%), but it is still unclear why this energetically highly inefficient process is preferred instead of OXPHOS (Koppenol et al., 2011). Although fermentation is less effective, it is ten times faster than full glucose oxidation, so it may support rapid ATP production to meet an increasing demand in intensively proliferating cancer cells (Devic, 2016; Liberti and Locasale, 2016). Furthermore, reducing OXPHOS may protect the cell from ROS produced by mitochondria during electron chain activity (Napolitano et al., 2021). Another reason for the preference for fermentation over OXPHOS may be associated with extracellular acidification due to the increased lactate that confers immune evasion ability onto cancer cells and creates a favorable TME (Boedtkjer and Pedersen, 2020; Chen et al., 2021). In accordance with this, we found that the lactate level was increased in the NOFs exposed to the EVs, but the difference was not statistically significant. The level of lactate in each individual sample revealed a large variation, which explain the lack of significance. This may be due to the cells having already secreted a large amount of lactate as part of a “reverse Warburg effect” (Jiang et al., 2019). In the reverse Warburg effect, stromal cells undergo aerobic glycolysis, but instead of using the produced intermediates such as lactate and pyruvate themselves, the cells secrete those energy metabolites to fuel cancer cells that have high energy demands due to their rapid proliferation (Pavlides et al., 2009). This finding is in line with the report of Zhang et al. (2020) which revealed that in co-culture with OSCC cells, NOFs undergo aerobic glycolysis, secrete lactate, and overexpress the lactate transporter MCT-4. This metabolically altered NOF phenotype possibly corresponds with impaired mitochondrial activity (Zhang et al., 2020), and may be an effect of EV cargo on recipient cells.

The present study revealed that in addition to pyruvate and lactate, several other metabolites involved in energy production, such as ATP, coenzyme A, dihydroxyacetone phosphate, UTP, NAD, FAD, cADPR, and docosahexaenoic acid, were dysregulated in NOFs following OSCC EVs exposure. Results of the metabolites detection with LC-MS were verified with Western blotting for PKM2 and ALDOA. Moreover, these findings are supported by the functional analysis demonstrating enrichment of glycolysis (related with pyruvate, ATP, dihydroxyacetone phosphate, and NAD), TCA cycle (related with pyruvate, ATP, coenzyme A, NAD, and FAD) and mitochondria electron transport chain (related with ATP, dihydroxyacetone phosphate, NAD, and FAD). Of interest, in addition to the established role of the TCA cycle in biosynthesis and bioenergetics, the TCA intermediates like α-ketoglutarate, fumarate, lactate, and succinate can affect various aspects of cancer progression or even act similarly to cytokines (Zasłona and O’Neill, 2020; Eniafe and Jiang, 2021). These metabolites were detected in the present study but were not significantly changed. However, even if not significant, disturbed levels of these metabolites could contribute to the overall picture of differences between control and OSCC EV-exposed fibroblasts. Furthermore, dysregulation of the TCA cycle and mitochondrial electron transport chain is considered another feature of the OSCC metabolic hallmark (Vitório et al., 2020; Bai et al., 2022). However, this finding may also be a consequence of the glycolysis dysregulation and Warburg effect (Jiang et al., 2019) rather than an independent event. Still, it is interesting that a single exposure to the OSCC EVs was sufficient to trigger substantial changes in NOFs.

Several fatty acids (FAs), such as nervonic acid, linoleate, oleate, palmitoleic acid, and docosahexaenoic acid, were significantly downregulated in NOFs exposed to OSCC EVs. Cancer cells redirect FAs from energy metabolism towards *de novo* lipogenesis to promote proliferation and lipid signaling (Louie et al., 2013). Nervonic acid plays a role in the immune response as it regulates transformation and proliferation of splenic lymphocytes, and yield of pro-inflammatory chemokines and cytokines (Borish and Steinke, 2003; Yamazaki et al., 2014; Yuan et al., 2023), which could affect the NOFs response to EV cargo and direct them towards a pro-tumorigenic phenotype. While oleate and linoleate stimulate proliferation in breast cancer (Yonezawa et al., 2004), oleate has anticancer effect in various other types of cancers (Carrillo et al., 2012). For example, in tongue squamous cell carcinoma, oleate induces apoptosis and autophagy (Jiang et al., 2017). Regardless of the role of FAs as pro- or anti-cancer agents (Denko, 2008; Jiang et al., 2017; Souza et al., 2017), it is possible that at the initial step of reprogramming toward a pro-tumorigenic phenotype of NOFs, detected downregulation of nervonic acid, linoleate, oleate, palmitoleic acid, and docosahexaenoic acid may rather reflect dysregulation of immune processes in fibroblasts as part of a defensive response to the OSCC EVs.

We also observed that the reaction of fibroblasts to OSCC EV exposure was time dependent. There was an early response already showing after 15 min, but the response was more pronounced after 24 h. However, at 48 h, the metabolic profile seemed to normalize in the EV-exposed fibroblasts. This may indicate that a single exposure to the molecular cargo carried by the EVs may not be sufficient to achieve a long-lasting change in metabolic status of NOFs. It is difficult to estimate the pattern of EV release during the course of cancer development and how frequent the stimulation of nearby cells is, but it can be expected that this is not an on-off phenomenon (Mathieu et al., 2021; Auber and Svenningsen, 2022). Therefore, it is reasonable to assume that normal cells subjected to long-term exposure to the cancer-derived EVs will undergo phenotype change, and that this probably will be a long-term effect, ultimately contributing to transformation of NOFs e.g., into CAFs.

EVs are carriers of the diversified molecules that can affect biological processes such as proliferation, apoptosis, DNA repair, angiogenesis, and immune response (Becker et al., 2016; Ciardiello et al., 2016; Søland et al., 2024). To date studies on OSCC EV profiling have mainly focused on the nucleic acid and protein cargo (Leung et al., 2021). In monocytes, the OSCC EV signature miR-21 and miR-27 led to activation of the inflammatory pathway, that in turn contributed to the establishment of a pro-inflammatory and protumorigenic milieu (Momen-Heravi and Bala, 2018). Such oncogenic miRNAs are considered as promising diagnostic markers for OSCC and other cancers (Li et al., 2016; Leung et al., 2021). On the other hand, more detailed multi-omic analysis of the OSCC EVs, covering also metabolomic and lipidomic data, revealed 11 hub proteins - candidates as prognostic markers (Busso-Lopes et al., 2021). Our current research revealed that OSCC EVs can affect NOF metabolome. Thus, the continuation of this study could be a similar analysis of the transcriptome and proteome to identify pathways that are affected by the OSCC. Such an approach could be beneficial to extend the knowledge on EV effects on normal cells and indicate potential targets for OSCC therapy. As a metabolomics profiling approach based on the LC-method and annotation by mass accuracy and LC retention times has its limitations, it could in future work be considered to introduce a labelled internal standard. It might be helpful to confirm significant results by validation of variations in sample preparation, extraction, and instrument performance and matrix effect. However, an internal standard needs to be selected carefully not to cause any ion suppression for analyzed metabolites. Therefore, more studies are needed to address all challenges associated with potential EV use in diagnosis, prognosis, and therapy of OSCC.

In summary, the present study identified a range of changes in NOF metabolites after exposure to OSCC-derived EVs. Various metabolites involved in glycolysis and amino acid and lipid metabolism, crucial for proper cell functioning, were affected. Nonetheless, it should be highlighted that the impact of cancer-derived EVs may go far beyond this and affect cell functioning at the level of transcriptome and proteome to induce changes that in the light of the obtained results can be considered as acquiring a pro-tumorigenic phenotype. Further studies are needed to identify molecular mechanisms involved in this phenotype switch. In the long-term perspective, a broad characterization of the effects of cancer-derived EVs on healthy cells should enable identification of potential biomarkers or targets for future anti-OSCC therapies.

## Data Availability

The data presented in the study are deposited in the Zenodo repository at https://zenodo.org/records/12667788.
